# The genome sequence of the Lesser Hornet Hoverfly, *Volucella inanis* (Linnaeus, 1758)

**DOI:** 10.12688/wellcomeopenres.18897.1

**Published:** 2023-02-10

**Authors:** Liam M. Crowley, Ryan Mitchell, University of Oxford and Wytham Woods Genome Acquisition Lab, Natural History Museum Genome Acquisition Lab, Darwin Tree of Life Barcoding collective, Wellcome Sanger Institute Tree of Life programme, Wellcome Sanger Institute Scientific Operations: DNA Pipelines collective, Tree of Life Core Informatics collective, Scarlett T. Weston, Karl R. Wotton, Darwin Tree of Life Consortium

**Affiliations:** 1 Department of Biology, University of Oxford, Oxford, UK; 2 Natural History Museum, London, UK; 3 Centre for Ecology and Conservation, University of Exeter, Penryn Campus, Penryn, Cornwall, UK

**Keywords:** Volucella inanis, lesser hornet hoverfly, genome sequence, chromosomal, Diptera

## Abstract

We present a genome assembly from an individual female *Volucella inanis* (the Lesser Hornet Hoverfly; Arthropoda; Insecta; Diptera; Syrphidae). The genome sequence is 961 megabases in span. Most of the assembly is scaffolded into six chromosomal pseudomolecules, including the assembled X sex chromosome. The mitochondrial genome has also been assembled and is 16.0 kilobases in length. Gene annotation of this assembly on Ensembl has identified 11,616 protein coding genes.

## Species taxonomy

Eukaryota; Metazoa; Ecdysozoa; Arthropoda; Hexapoda; Insecta; Pterygota; Neoptera; Endopterygota; Diptera; Brachycera; Muscomorpha; Syrphoidea; Syrphidae; Eristalinae; Volucellini; *Volucella*; *Volucella inanis* (Linnaeus, 1758) (NCBI:txid226151).

## Background

**Figure 1.**
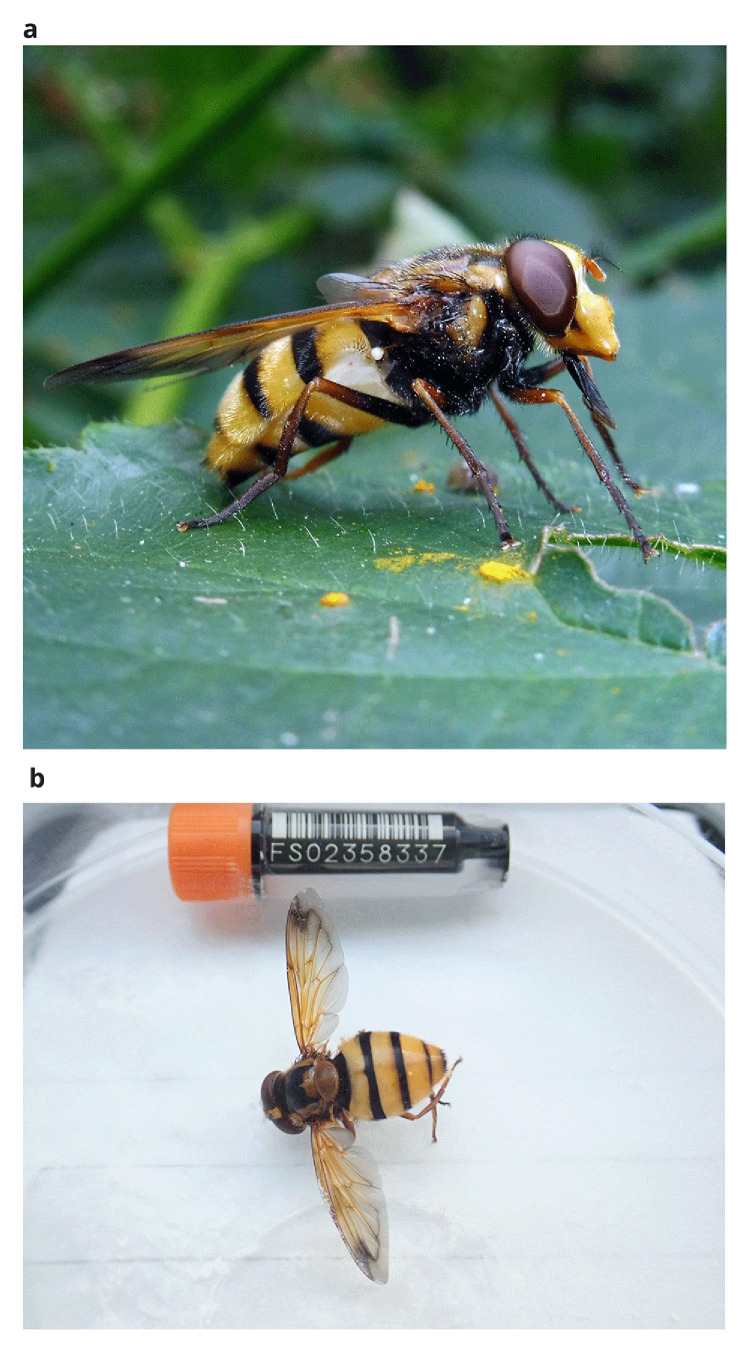
*Volucella inanis.* **a**) Photograph of *Volucella inanis* by Barry Walter (used under CC-BY 4.0 license). **b**) Photograph of the *Volucella inanis* (idVolInan1) specimen used for genome sequencing.

The Wasp Plumehorn or Lesser Hornet hoverfly, *Volucella inanis*, ( Figure 1a) is a large hoverfly, whose distinct yellow and black markings give it a wasp-like appearance. Although it is similar in appearance to the Hornet hoverfly ( *Volucella zonaria*), the smaller-sized *V. inanis* can be identified as the markings at the base of the abdomen are yellow rather than chestnut-coloured, and the yellow colouration to the sternites is greater ( Ball & Morris, 2015). *V. inanis* is noted to be a Batesian mimic of the European hornet ( *Vespa crabro*) and the Common wasp ( *Vespula vulgaris*), whose aposematic colours reduce the risk of predation ( Hlaváček *et al.*, 2022; Parmentier, 2020).

This hoverfly is distributed across the eastern Palearctic, including most of central and southern Europe, Syria and North Africa, although its range is expanding northwards ( Doyle *et al.*, 2020; Rupp, 1989). Formerly restricted to the southeast in the UK, it has undergone a rapid range expansion over recent decades and can now be found northwards to Yorkshire ( Ball & Morris, 2015). Adults are abundant mid- to late summer in woodland edges or green city spaces, feeding from the flowers of Buddleja, brambles, thistles, umbellifers, ivy, Snowberry and Devil's-bit scabious ( Ball & Morris, 2015; Stubbs & Falk, 2002). Females are often associated with the nests of the German wasp ( *Vespula germanica*), Common wasp ( *Vespula vulgaris*) and European hornet ( *Vespa crabro*), attempting to lay eggs undetected near the nest entrances ( Bothe, 1984; Speight, 2011). Upon hatching ~5 days later, the *V. inanis* larvae will invade the host’s nest ( Bothe, 1984). These ectoparasitic larvae are very flattened, distinguishing them from other *Volucella* sp., and allowing them to fit inside the larval cells of their host, alongside the social wasps’ larva ( Parmentier, 2020). The *V. inanis* larva first scavenges on the excretions of the developing wasp larva. However, once the wasp larva pupates, *V. inanis* consumes the whole organism ( van Veen, 2004), later leaving the nest and pupating underground.

The sequencing of this high-quality genome, accomplished for the first time through the Darwin Tree of Life project, will help uncover a greater understanding of the fascinating biology and ecology of this species. This includes studies investigation the evolution of mimicry and ectoparasitism, the biogeographical impacts of climate change and conservation of important pollinator species. 

## Genome sequence report

The genome was sequenced from one female *V. inanis* specimen ( Figure 1) collected from Wytham Great Wood (51.773, –1.333). A total of 26-fold coverage in Pacific Biosciences single-molecule HiFi long reads and 40-fold coverage in 10X Genomics read clouds were generated. Primary assembly contigs were scaffolded with chromosome conformation Hi-C data. Manual assembly curation corrected 212 missing joins or mis-joins, and removed 4 haplotypic duplications, reducing the scaffold number by 71.43% and increasing the scaffold N50 by 63.95%.

**Figure 2.**
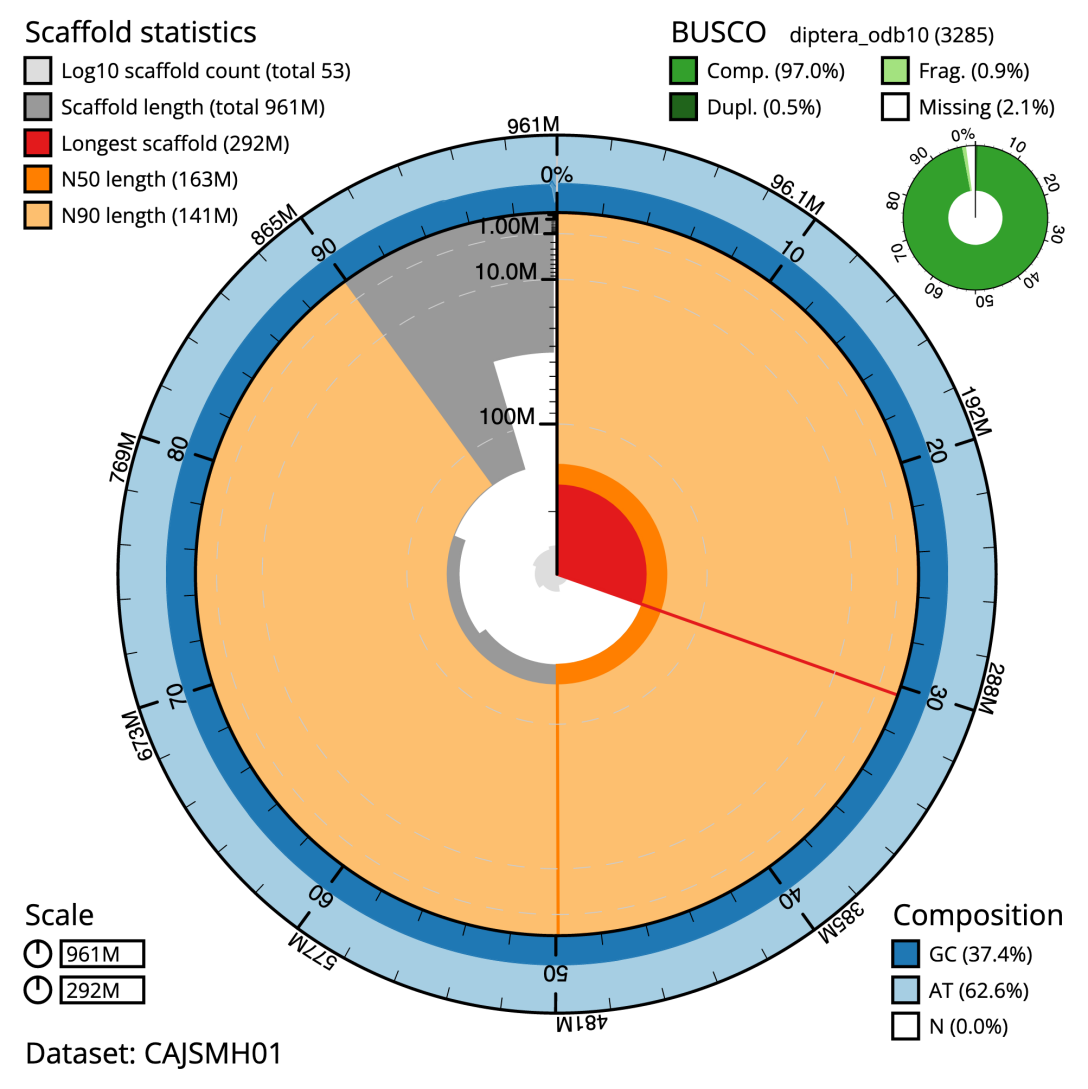
Genome assembly of *Volucella inanis*, idVolInan1.1: metrics. The BlobToolKit Snailplot shows N50 metrics and BUSCO gene completeness. The main plot is divided into 1,000 size-ordered bins around the circumference with each bin representing 0.1% of the 961,462,834 bp assembly. The distribution of scaffold lengths is shown in dark grey with the plot radius scaled to the longest scaffold present in the assembly (292,306,469 bp, shown in red). Orange and pale-orange arcs show the N50 and N90 scaffold lengths (163,465,417 and 141,167,267 bp), respectively. The pale grey spiral shows the cumulative scaffold count on a log scale with white scale lines showing successive orders of magnitude. The blue and pale-blue area around the outside of the plot shows the distribution of GC, AT and N percentages in the same bins as the inner plot. A summary of complete, fragmented, duplicated and missing BUSCO genes in the diptera_odb10 set is shown in the top right. An interactive version of this figure is available at https://blobtoolkit.genomehubs.org/view/idVolInan1.1/dataset/CAJSMH01/snail.

The final assembly has a total length of 961.4 Mb in 52 sequence scaffolds with a scaffold N50 of 163.5 Mb ( Table 1). Most (99.92%) of the assembly sequence was assigned to six chromosomal-level scaffolds, representing five autosomes and the X sex chromosome. Chromosome-scale scaffolds confirmed by the Hi-C data are named in order of size ( Figure 2– Figure 5; Table 2). The assembly has a BUSCO v5.2.2 ( Manni *et al.*, 2021) completeness of 97% (single 96.5%, duplicated 0.5%) using the diptera_odb10 reference set ( *n* = 3,285). While not fully phased, the assembly deposited is of one haplotype. Contigs corresponding to the second haplotype have also been deposited.

**Table 1.** Genome data for *Volucella inanis,* idVolInan1.1.

Project accession data	
Assembly identifier	idVolInan1.1
Species	*Volucella inanis*
Specimen	idVolInan1
NCBI taxonomy ID	226151
BioProject	PRJEB44982
BioSample ID	SAMEA7520171
Isolate information	male
Raw data accessions	
PacificBiosciences SEQUEL II	ERR6406209, ERR6635598, ERR6635599
10X Genomics Illumina	ERR6054739–ERR6054742
Hi-C Illumina	ERR6054743, ERR6054744
PolyA RNA-Seq Illumina	ERR6464927
Genome assembly	
Assembly accession	GCA_907269105.1
*Accession of alternate haplotype*	GCA_907269115.1
Span (Mb)	961.4
Number of contigs	405
Contig N50 length (Mb)	30.1
Number of scaffolds	52
Scaffold N50 length (Mb)	163.5
Longest scaffold (Mb)	292.3
BUSCO * completeness	C:97.0%[S:96.5%,D:0.5%], F:0.9%,M:2.1%,n:3,285
Genome annotation	
Number of protein-coding genes	11,616
Non-coding genes	1,167
Number of transcripts	19,061

* BUSCO scores based on the diptera_odb10 BUSCO set using v5.2.2. C = complete [S = single copy, D = duplicated], F = fragmented, M = missing, n = number of orthologues in comparison. A full set of BUSCO scores is available at https://blobtoolkit.genomehubs.org/view/idVolInan1.1/dataset/CAJSMH01/busco.

**Figure 3.**
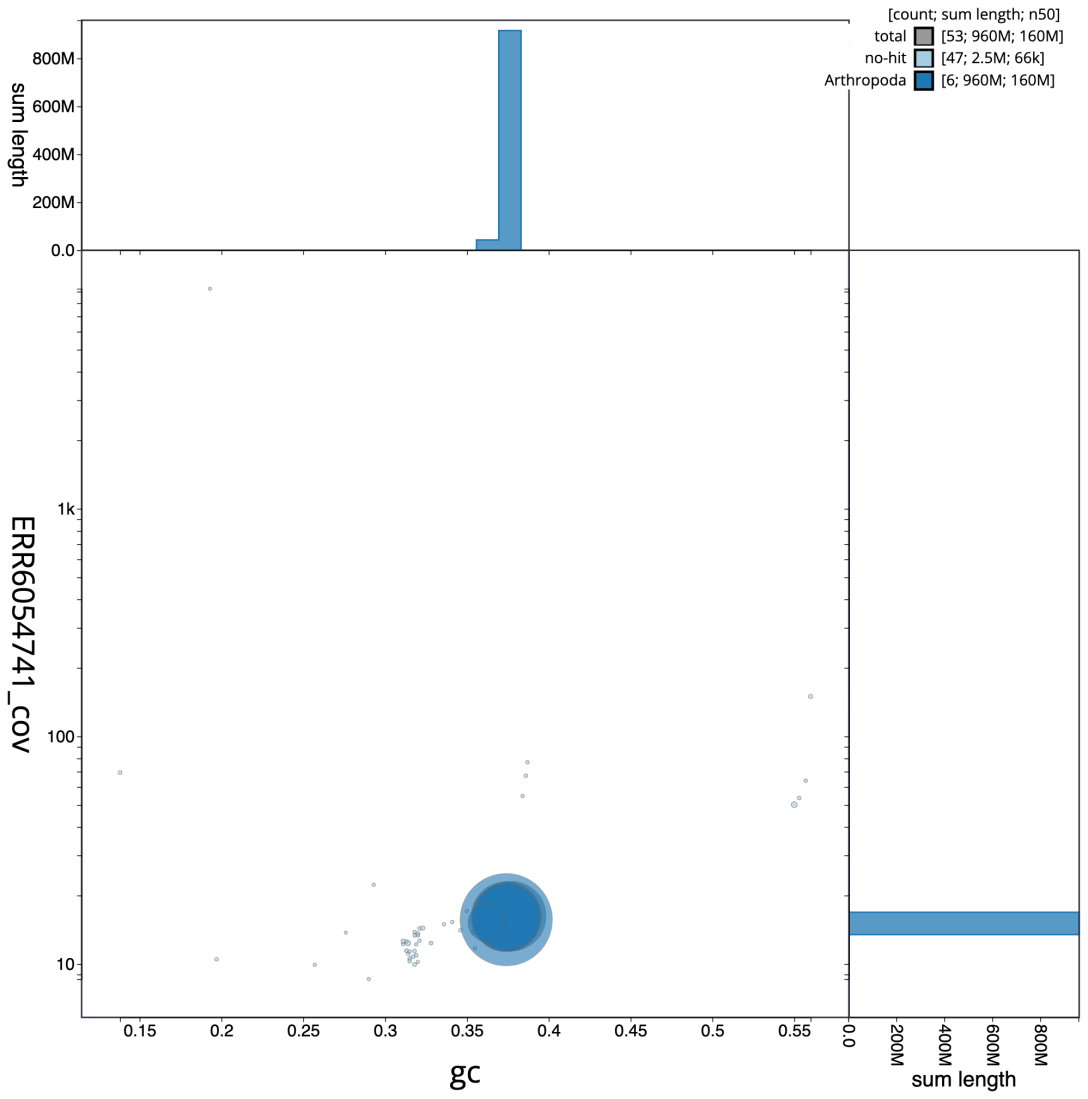
Genome assembly of *Volucella inanis*, idVolInan1.1: GC coverage. BlobToolKit GC-coverage plot. Scaffolds are coloured by phylum. Circles are sized in proportion to scaffold length. Histograms show the distribution of scaffold length sum along each axis. An interactive version of this figure is available at https://blobtoolkit.genomehubs.org/view/idVolInan1.1/dataset/CAJSMH01/blob.

**Figure 4.**
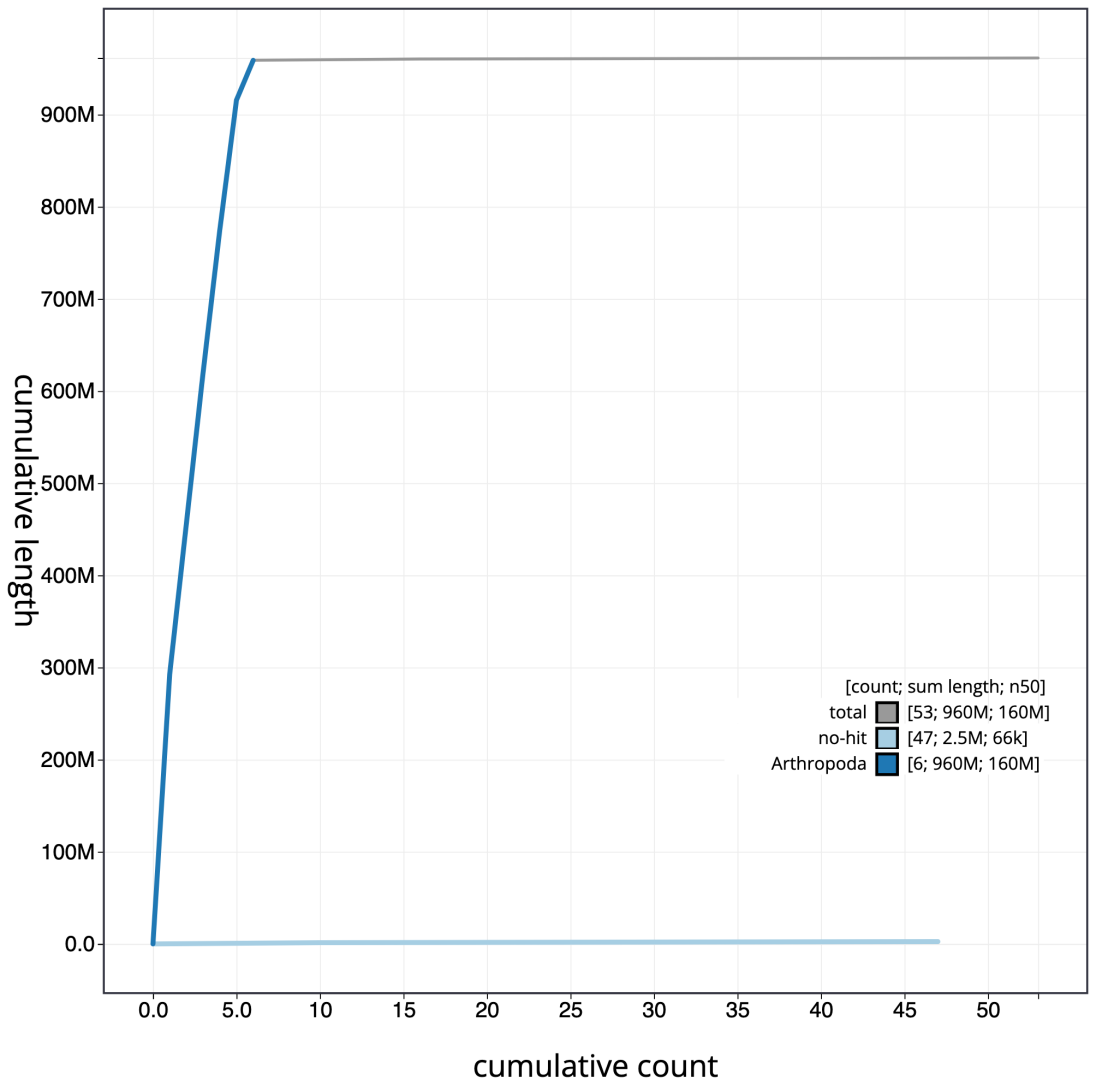
Genome assembly of *Volucella inanis*, idVolInan1.1: cumulative sequence. BlobToolKit cumulative sequence plot. The grey line shows cumulative length for all scaffolds. Coloured lines show cumulative lengths of scaffolds assigned to each phylum using the buscogenes taxrule. An interactive version of this figure is available at https://blobtoolkit.genomehubs.org/view/idVolInan1.1/dataset/CAJSMH01/cumulative.

**Figure 5.**
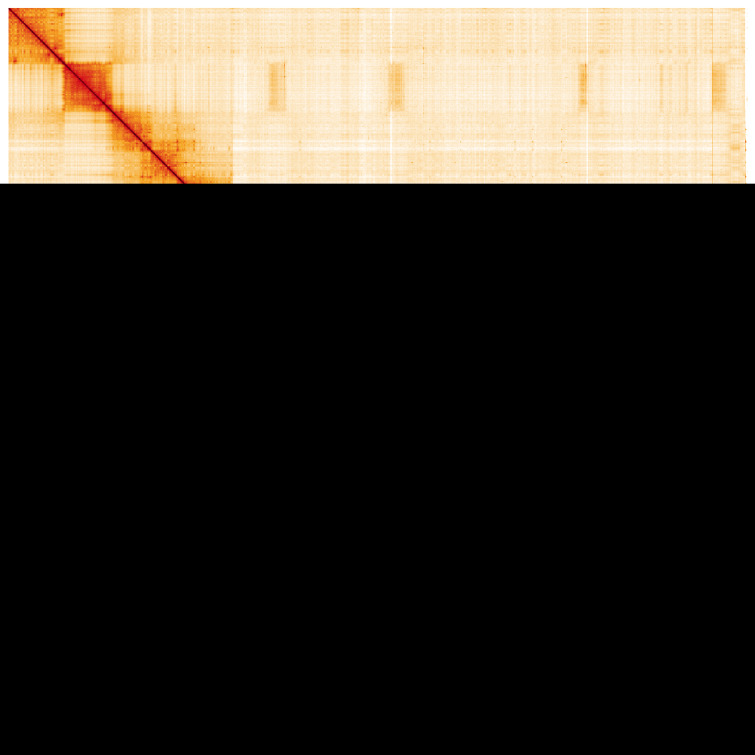
Genome assembly of *Volucella inanis*, idVolInan1.1: Hi-C contact map. Hi-C contact map of the idVolInan1.1 assembly, visualised using HiGlass. Chromosomes are shown in order of size from left to right and top to bottom. An interactive version of this figure may be viewed at https://genome-note-higlass.tol.sanger.ac.uk/app/?config=O-m3ajxFT8ugcnrEilKjLQ.

## Genome annotation report

The *V. inanis* (idVolInan1) genome assembly was annotated using the Ensembl rapid annotation pipeline ( Table 1; https://rapid.ensembl.org/Volucella_inanis_GCA_907269105.1/). The resulting annotation includes 19,061 transcribed mRNAs from 11,616 protein-coding and 1,167 non-coding genes.

**Table 2.** Chromosomal pseudomolecules in the genome assembly of *Volucella inanis*, idVolInan1.

INSDC accession	Chromosome	Size (Mb)	GC%
OU026153.1	1	292.31	37.4
OU026155.1	2	163.47	37.7
OU026154.1	3	164.05	37.4
OU026156.1	4	154.76	37.4
OU026157.1	5	141.17	37.4
OU026158.1	X	43.18	36.2
OU026159.1	MT	0.02	19.3
-	unplaced	2.52	37.6

## Methods

### Sample acquisition and nucleic acid extraction

A male *Volucella inanis* specimen (idVolInan1) was caught using a net in Wytham Great Wood, Oxfordshire (biological vice-county: Berkshire), UK (latitude 51.773, longitude –1.333) on 13 August 2019. A second specimen (idVolInan2) was collected at Hever Castle, Kent, UK (latitude 51.19, longitude 0.12) on 27 August 2020. Specimens idVolInan1 and idVolInan2 were collected and identified by Liam Crowley (University of Oxford). A third specimen (idVolInan3) was collected in Hartslock Reserve, Oxfordshire (latitude 51.51, longitude –1.11) by Ryan Mitchell (Natural History Museum). All specimens were preserved on dry ice.

DNA was extracted at the Tree of Life laboratory, Wellcome Sanger Institute (WSI). The idVolInan1 sample was weighed and dissected on dry ice with tissue set aside for Hi-C sequencing. Head and thorax tissue was disrupted using a Nippi Powermasher fitted with a BioMasher pestle. High molecular weight (HMW) DNA was extracted using the Qiagen MagAttract HMW DNA extraction kit. Low molecular weight DNA was removed from a 20 ng aliquot of extracted DNA using 0.8X AMpure XP purification kit prior to 10X Chromium sequencing; a minimum of 50 ng DNA was submitted for 10X sequencing. HMW DNA was sheared into an average fragment size of 12–20 kb in a Megaruptor 3 system with speed setting 30. Sheared DNA was purified by solid-phase reversible immobilisation using AMPure PB beads with a 1.8X ratio of beads to sample to remove the shorter fragments and concentrate the DNA sample. The concentration of the sheared and purified DNA was assessed using a Nanodrop spectrophotometer and Qubit Fluorometer and Qubit dsDNA High Sensitivity Assay kit. Fragment size distribution was evaluated by running the sample on the FemtoPulse system.

RNA was extracted from thorax tissue of idVolInan3 in the Tree of Life Laboratory at the WSI using TRIzol, according to the manufacturer’s instructions. RNA was then eluted in 50 μl RNAse-free water and its concentration assessed using a Nanodrop spectrophotometer and Qubit Fluorometer using the Qubit RNA Broad-Range (BR) Assay kit. Analysis of the integrity of the RNA was done using Agilent RNA 6000 Pico Kit and Eukaryotic Total RNA assay.

### Sequencing

Pacific Biosciences HiFi circular consensus and 10X Genomics read cloud DNA sequencing libraries were constructed according to the manufacturers’ instructions. Poly(A) RNA-Seq libraries were constructed using the NEB Ultra II RNA Library Prep kit. DNA and RNA sequencing were performed by the Scientific Operations core at the WSI on Pacific Biosciences SEQUEL II (HiFi), Illumina HiSeq 4000 (RNA-Seq) and HiSeq X Ten (10X) instruments. Hi-C data were also generated from head and thorax tissue of idVolInan2 using the Arima v2 kit and sequenced on the Illumina NovaSeq 6000 instrument.

### Genome assembly

Assembly was carried out with Hifiasm ( Cheng *et al.*, 2021) and haplotypic duplication was identified and removed with purge_dups ( Guan *et al.*, 2020). One round of polishing was performed by aligning 10X Genomics read data to the assembly with Long Ranger ALIGN, calling variants with freebayes ( Garrison & Marth, 2012). The assembly was then scaffolded with Hi-C data ( Rao *et al.*, 2014) using SALSA2 ( Ghurye *et al.*, 2019). The assembly was checked for contamination and corrected using the gEVAL system ( Chow *et al.*, 2016) as described previously ( Howe *et al.*, 2021). Manual curation was performed using gEVAL, HiGlass ( Kerpedjiev *et al.*, 2018) and Pretext ( Harry, 2022). The mitochondrial genome was assembled using MitoHiFi ( Uliano-Silva *et al.*, 2022), which performed annotation using MitoFinder ( Allio *et al.*, 2020). The genome was analysed and BUSCO scores generated within the BlobToolKit environment ( Challis *et al.*, 2020). Table 3 contains a list of all software tool versions used, where appropriate.

**Table 3.** Software tools and versions used.

Software tool	Version	Source
BlobToolKit	3.0.5	Challis *et al.*, 2020
freebayes	1.3.1-17-gaa2ace8	Garrison & Marth, 2012
gEVAL	N/A	Chow *et al.*, 2016
Hifiasm	0.12	Cheng *et al.*, 2021
HiGlass	1.11.6	Kerpedjiev *et al.*, 2018
Long Ranger ALIGN	2.2.2	https://support.10xgenomics.com/genome-exome/ software/pipelines/latest/advanced/other-pipelines
MitoHiFi	2	Uliano-Silva *et al.*, 2022
PretextView	0.2	Harry, 2022
purge_dups	1.2.3	Guan *et al.*, 2020
SALSA	2.2	Ghurye *et al.*, 2019

### Genome annotation

The Ensembl gene annotation system ( Aken *et al.*, 2016) was used to generate annotation for the *V. inanis* assembly (GCA_907269105.1). Annotation was created primarily through alignment of transcriptomic data to the genome, with gap filling via protein to-genome alignments of a select set of proteins from UniProt ( UniProt Consortium, 2019).

### Ethics/compliance issues

The materials that have contributed to this genome note have been supplied by a Darwin Tree of Life Partner. The submission of materials by a Darwin Tree of Life Partner is subject to the Darwin Tree of Life Project Sampling Code of Practice. By agreeing with and signing up to the Sampling Code of Practice, the Darwin Tree of Life Partner agrees they will meet the legal and ethical requirements and standards set out within this document in respect of all samples acquired for, and supplied to, the Darwin Tree of Life Project. Each transfer of samples is further undertaken according to a Research Collaboration Agreement or Material Transfer Agreement entered into by the Darwin Tree of Life Partner, Genome Research Limited (operating as the Wellcome Sanger Institute), and in some circumstances other Darwin Tree of Life collaborators.

## Data Availability

European Nucleotide Archive: *Volucella inanis* (lesser hornet hoverfly). Accession number PRJEB44982; https://identifiers.org/ena.embl/PRJEB44982 ( Wellcome Sanger Institute, 2021). The genome sequence is released openly for reuse. The *Volucella inanis* genome sequencing initiative is part of the Darwin Tree of Life (DToL) project. All raw sequence data and the assembly have been deposited in INSDC databases. Raw data and assembly accession identifiers are reported in Table 1.

## Author information

Members of the University of Oxford and Wytham Woods Genome Acquisition Lab are listed here: https://doi.org/10.5281/zenodo.4789929.

Members of the Natural History Museum Genome Acquisition Lab are listed here: https://doi.org/10.5281/zenodo.4790043.

Members of the Darwin Tree of Life Barcoding collective are listed here: https://doi.org/10.5281/zenodo.4893703.

Members of the Wellcome Sanger Institute Tree of Life programme are listed here: https://doi.org/10.5281/zenodo.4783585.

Members of Wellcome Sanger Institute Scientific Operations: DNA Pipelines collective are listed here: https://doi.org/10.5281/zenodo.4790455.

Members of the Tree of Life Core Informatics collective are listed here: https://doi.org/10.5281/zenodo.5013541.

Members of the Darwin Tree of Life Consortium are listed here: https://doi.org/10.5281/zenodo.4783558.
