# Supplementary material for: The genome sequence of the Lesser Hornet Hoverfly, Volucella inanis (Linnaeus, 1758)
Source: Wellcome Open Res. 2023 Feb 10;8:69. [Version 1] doi: 10.12688/wellcomeopenres.18897.1 (PMC10620480; doi:10.12688/wellcomeopenres.18897.1)
Supplement: Peer review history

# Reviewer report: Reviewer response for version 1

**DOI:** 10.21956/wellcomeopenres.20954.r68817

**Published:** 2023-11-01

**Authors:** C. Scott Clem

**Affiliations:** 1 University of Georgia, Athens, Georgia, USA

This paper presents a genome assembly of the hover fly species Vollucella inanis. I think the manuscript is well-written, it is thorough, and I see no need for any major changes. I am excited to see what projects develop from this genome.

 I am wondering if the authors may have additional metadata they could provide on the specimens. What type of habitat were they collected in? Also, which flowers were they visiting? Are the second and third specimens female or male?

Are sufficient details of methods and materials provided to allow replication by others?

Yes

Is the rationale for creating the dataset(s) clearly described?

Yes

Are the datasets clearly presented in a useable and accessible format?

Yes

Are the protocols appropriate and is the work technically sound?

Yes

Reviewer Expertise:

I am an entomologist and ecologist with a specialty in syrphids. I have never published a genome previously, but I am familiar with some of the methodologies and protocols. My knowledge regarding these types of papers, however, is limited.

I confirm that I have read this submission and believe that I have an appropriate level of expertise to confirm that it is of an acceptable scientific standard.

# Reviewer report: Reviewer response for version 1

**DOI:** 10.21956/wellcomeopenres.20954.r68815

**Published:** 2023-11-01

**Authors:** Luis Diambra

**Affiliations:** 1 National University of La Plata, Argentina, Argentina

This manuscript from Crowley et al. reports the genome draft of the Diptera V. inanis. Data generation and analysis, used for several other genomes recently sequenced by the Tree of Life Project, are clearly explained and contain enough information to interpret this important genomic resource. Overall this is a quality paper and merits approval for indexing.

Are sufficient details of methods and materials provided to allow replication by others?

Yes

Is the rationale for creating the dataset(s) clearly described?

Yes

Are the datasets clearly presented in a useable and accessible format?

Yes

Are the protocols appropriate and is the work technically sound?

Yes

Reviewer Expertise:

Bioinformatics

I confirm that I have read this submission and believe that I have an appropriate level of expertise to confirm that it is of an acceptable scientific standard.

# Reviewer report: Reviewer response for version 1

**DOI:** 10.21956/wellcomeopenres.20954.r62112

**Published:** 2023-08-10

**Authors:** Eric Aguiar

**Affiliations:** 1 Universidade Estadual de Santa Cruz, Ilhéus, State of Bahia, Brazil

In this manuscript, Crowley and colleagues describe the genome of the Lesser Hornet Hoverfly (V *olucella inanis). *The paper is well-written, with appropriate methods containing all the information necessary to interpret the data. Of note, the genome of this hoverfly is a valuable resource that can give, for instance, insights into the ectoparasite behavior of the *V. inanis *larvae in parasitizing larvae of other species.

 Therefore, I recommend the publication of the manuscript in its current form.

Are sufficient details of methods and materials provided to allow replication by others?

Yes

Is the rationale for creating the dataset(s) clearly described?

Yes

Are the datasets clearly presented in a useable and accessible format?

Yes

Are the protocols appropriate and is the work technically sound?

Yes

Reviewer Expertise:

Bioinformatics; Host-Pathogen Interactions; Genomics

I confirm that I have read this submission and believe that I have an appropriate level of expertise to confirm that it is of an acceptable scientific standard.
